# Omega and heart rate variability in overweight and obese schoolchildren

**DOI:** 10.1038/s41390-025-03913-5

**Published:** 2025-02-19

**Authors:** Hoda Atef Abdelsattar Ibrahim, Rodina Sobhi, Nouran Khaled Farouk, Faten Mohamed AbdelAziz

**Affiliations:** 1 Lecturer, Pediatric Clinical Nutrition Unit, Department of Pediatrics, Faculty of Medicine, Cairo University, Cairo 12613, Giza, 11562 Egypt; 2 Department of Pediatrics, Faculty of Medicine, Cairo University, Cairo 12613, Giza, 11562 Egypt

## Abstract

**Background:**

Overweight and obese children are risky for developing chronic cardiovascular disorders. Heart rate variability is a valuable indicator of the cardiovascular health.

**Aim:**

To outline the impact of supplementation of omega-3 on the variability of the heart rate.

**Methods:**

A randomized interventional control study has been done on 60 overweight and obese children; 30 children were assigned to the interventional group who received omega-3 with the standard recommendations and 30 children were assigned to the control group who received the standard recommendations only. Resting 10 min Holter ECG was done at the start and after 3 months of omega-3 supplementation in both groups to observe and compare the difference in the heart rate variability. In addition, lipid profiles were investigated and compared.

**Results:**

Difference in measures of the heart rate variability; RMSSD, SDNN, and pNN50 were increased significantly in cases compared to the controls (*P* value = 0.017, 0.009, and 0.043 respectively). Differences in measures of the lipid profiles differed only in triglycerides which decreased significantly in cases and HDL which increased significantly in the cases (*P* value = 0.006, 0.005 respectively).

**Conclusion:**

Our results suggested that omega-3 supplementation might improve cardiovascular health in overweight and obese children.

**Impact:**

- This study discloses the importance of supplementation of omega-3 fatty acids as a cardioprotective dietary supplement.
- Omega-3 fatty acids can improve heart rate variability in overweight and obese children.
- Omega-3 fatty acids can also improve the lipid profiles in overweight and obese children.

## Introduction

Overweight and obesity are common health concerns in the pediatric age group. These vulnerable children are at increased probability of being overweight as adults and acquiring many cardiovascular disorders. Despite the evident link between higher BMI (body mass index) and developing cardiovascular diseases, the causes for such correlation are unclear. The relationship between dietary fats and pediatric obesity has been a long-standing debate. Clinical studies show that trans fatty acids can lead to increased insulin resistance. The overweight and obese children show higher intakes of these trans fatty acids, thus they are vulnerable to insulin resistance.^1^

In addition to insulin resistance, it has been proposed that cardiovascular illnesses can include several disorders such as worsening lipid profiles, and autonomic cardiac control.^2,3^

Heart rate variability (HRV) is a measure of autonomic effects on heart rate. It has frequently been utilized as a trans-situationally consistent biomarker for cardiovascular health and emotional or cognitive processes. It has been shown that the stability of heart rate variability indices reflects the parasympathetic activity^4^

Childhood obesity is linked to unfavorable lipid profiles, indicating that overweight and obese children should be screened. This is attributed to the abnormal lipid profiles, especially triglycerides, HDL, and LDL cholesterol levels.

Recent research has found that omega-3 improves these parameters. Omega-3 has been revealed in studies to lower blood triglyceride levels and improve insulin sensitivity, and variability of the heart rate measures, all of which are potential indicators of children’s cardiovascular health.^5–7^

As a result, supplementation of omega-3 may be a low-cost strategy with fewer adverse outcomes for overweight and obese children, perhaps slowing the progression of chronic cardiovascular illness.^8–10^

In this study, we examined how omega-3 supplementation affected the variability of the heart rate in overweight and obese school children. In addition, the impact of omega-3 on their lipid profiles was another core of interest in our study.

## Methods

### Study design and setting

Our study is a parallel randomized interventional control trial on overweight and obese children with simple obesity who were following at the general clinic at Children’s Hospital Cairo University (CHCU) during the period from April 2024 to September 2024.

### Inclusion and exclusion criteria

Children aged from 5–12 years of both sexes with simple obesity whose parents or caregivers agreed to be enrolled in the study were included. Children known to have cardiac diseases, relevant medications affecting the heart rate, endocrinal, genetic, or other causes of secondary obesity were excluded.

### Population, intervention, comparison, and outcome (PICO)^11^

#### Population

Schoolchildren with obesity and overweight who were following at the general clinic at Children’s Hospital Cairo University (CHCU)

#### Intervention

Impact of omega 3 on time domain heart rate variability and lipid profiles

#### Comparison

The interventional and control groups were compared regarding differences in RMSSD, SDNN,pNN50%, and lipid profiles, including total cholesterol, LDL cholesterol, HDL cholesterol, and triglycerides.

#### Outcome

Changes in time domain heart rate variability and levels of lipid profiles.

### Data collected and intervention

**Fig. 1:**
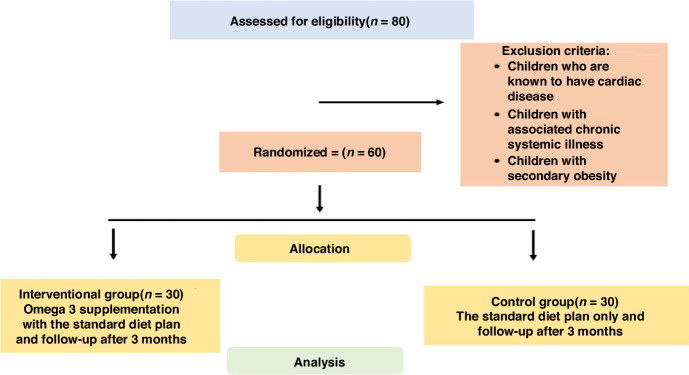
Flow chart of the study.

Informed written consent was obtained from parents/legal guardians before enrollment in the study. All children in this study were enrolled to the full history including demographic data, and present and past history as causes of obesity to exclude those with secondary causes. Additionally, clinical data including anthropometric data such as BMI, laboratory data as lipid profiles, and the variability of the heart rates were collected at the start of the study and after omega intervention to the intervention group (400 mg eicosapentaenoic acid (EPA) and 200 mg docosahexaenoic acid (DHA) daily for 3 months was taken) and the control group. Although there are no settled recommendations for the advisable doses of EPA and/or DHA for children above 2 years of age, we tried to optimize the ratio of EPA to DHA to 2:1 as this ratio was found to decrease the cardiovascular risk. This dose of DHA has been found to significantly increase erythrocyte and plasma phospholipid DHA levels,^3,12–16^ Figure 1 shows the flow chart of the study.

### Case definition

1. Resting heart rate variability:^17^ Time-domain indices of heart rate variability compute measures of the variability in the inter-beat interval:

A. RMSSD (ms):Root mean square of successive RR interval differences.
B. SDNN ms :Standard deviation of NN intervals (Time (normalized) between two detected heartbeat detections, calculated for every QRS event).
C. pNN50% :Percentage of successive RR intervals that differ by more than 50 ms.

### Holter ECG

#### Set up

Holter ECG (schiller MT 200) was attached and then the children were placed in a supine position. Supine position has demonstrated higher reliability heart rate variability measures in children than standing or sitting. Then, heart rate variability was recorded for 10 min at a sampling frequency of 1000 Hz. Children were encouraged and informed to keep relaxed, and not speak or move at the time of evaluation.

In the time domain, we calculated the squared root of the mean of the sum of the squares of consecutive normal R-R interval differences (RMSSD) and the percentage number of pairs of adjacent normal R-R intervals deviating by more than 50 ms in the whole recording (pNN50) as (physical activity) indexes. In addition, we calculated the standard deviation for all normal R-R intervals (SDNN). The follow-up was after an average of 3 months.

2. Definitions of overweight and obesity^18–20^

A. Overweight is defined as a BMI over the 85th percentile for age or a BMI Z score more than 1 SD above the WHO Child Standards for BMI 5–19 years.
B. Obesity is defined as a BMI over the 97th percentile for age or a BMI Z score above 2 SD of the WHO Child Standards for BMI 5–19 years.

3. Lipid Profiles: lipid profiles included in the study are HDL, LDL cholesterol, total cholesterol, and triglycerides. These labs were withdrawn from the enrolled children in the morning after 6–8 h of sleep to remove any postprandial bias.

### Randomization and control of other potential bias

An independent investigator used a computerized program (GraphPad QuickCalcs) to randomly assign the recruited children to either omega-3 or control in a 1:1 ratio. The intervention was concealed from the investigator, attending physicians, children, and parents. The randomization sequence was only unlocked once the research was completed, and the data was anonymously stored and analyzed using encryption codes.

### Ethical committee approval

The protocol was revised and accepted by the Research Committee of the Pediatrics Department, Faculty of Medicine, Cairo University; the ethical committee approval was provided by the Ethics Committee, Faculty of Medicine, Cairo University(IRB number :MS-547-2023). The study posed no harm or injury to the enrolled children. Omega-3 as a dietary supplement is regarded as safe in the recommended doses. We also performed our research under the Declaration of Helsinki.

#### Clinical trial registration

The protocol of this study was revised and registered on clinicaltrials.gov;Identifier: https://clinicaltrials.gov/study/NCT06555705.

#### Sample size

The primary outcome was to detect the effect of omega-3 fatty acid supplementation on variability of the heart rate. Baumann et al estimated the heart rate variability by measuring the standard deviation (SD) of the normal-to-normal (SDNN) inter-beat intervals, root mean square of the successive differences (RMSSD) between the normal heartbeats and percentage of successive RR intervals (pNN50). Root mean square of successive differences (pNN50) was found to be 10.2 ± 8.3% at baseline and 18.5 ± 13.6% after omega-3 fatty acid supplementations.^3^ By using the G*Power software (version 3.1.9.2), the following criteria were considered for sample size calculation: Confidence level 95%, margin of error 5% (type 1 error 0.05), power of the study 80% (type 2 error 0.20). The minimum required sample size for the study is 29 for each group.

### Statistical analysis

Numerical data were statistically described in terms of mean; standard deviation (SD), median, and interquartile range (IQR) according to the normality tests ; Kolmogorov–Smirnov test and the Shapiro–Wilk test. Categorical variables were described as frequencies (number of cases) and percentages when appropriate. The normally distributed numerical data were compared using *T* test (independent). Regarding data not normally distributed, a comparison of numerical variables was done using the Mann-Whitney U test. Two-sided *p* values less than or equal to 0.05 were considered statistically significant. The Spearman correlation was done between non-parametric numerical variables. Univariable and multivariable regressions were done to control the basal lipid profiles as possible cofounders in the improvement of laboratory changes.

All statistical analyses were carried out using the computer program IBM SPSS (Statistical Package for the Social Science; IBM Corp, Armonk, NY, US) release 25 for Microsoft Windows. JASP 0.17.3.0 software was used for the mediation analysis.

## Results

Our study enrolled sixty overweight and obese schoolchildren who were attending the general clinics at Children’s Hospital Cairo University from April 2024 to September 2024. We aimed to assess the impact of omega-3 supplements on the variability of the heart rate in these overweight and obese children. Males predominated in the current study. The sociodemographic and clinical criteria of all study participants are described in Table 1. The clinical criteria of each of the interventional and control groups are illustrated in Table 2. As illustrated, no significant difference was found between cases and controls before supplementation of Omega-3 except for total cholesterol and triglycerides (which were significantly higher in cases) and HDL (which were significantly higher in controls). Despite these significant differences, the difference for each group favored the interventional group (cases)as illustrated in Table 4.

**Table 1:** Sociodemographic and clinical criteria of the study participants.

Variables	Results
**Age**	
Median (IQR)	9.1
Min-Max	5.1–12.2
**Males and females distribution**	
Males: N (%)	39 (65%)
Females: N (%)	21 (35%)
**Weight in kg:** Median (IQR)	55.7 (19)
Min-Max	28–71
**Weight Z score;** Mean (SD)	2.8 (0.46)
Min-Max	1.3–3.6
**Height in meters;** Median (IQR)	1.3 (0.23)
Min-Max	1.1–1.5
**Height Z score;** Mean (SD)	0.6 (0.4)
Min-Max	−0.41–1.8
**BMI;** Median (IQR)	29.9 (2.14)
Min-Max	23–32
**BMI Z score;** Mean (SD)	2.5 (0.53)
Min-Max	1.6–3.6
**Total cholesterol;** Median (IQR)	175 (27)
Min-Max	102–199
**LDL -cholesterol;** Median (IQR)	104.5 (57.7)
Min-Max	52–140
**HDL cholesterol;** Median (IQR)	44 (10)
Min-Max	27–56
**Triglycerides;** Mean (SD)	94.3 (19.1)
Min-Max	62–139

*IQR* interquartile range, *Min* minimum, *Max* maximum, *SD* standard deviation, *BMI* body mass index.

**Table 2:** Clinical criteria of the interventional and control groups.

	Interventional (Cases)	Control	*P* value
**Matching cases with controls at the start of the study (before Omega-3 supplementation)**			
RMSSD			
Median (IQR)	30(33)	36(27)	0.76^(1)^
Min-Max	7–148	11–218	
SDDN			
Median (IQR)	45.5(34)	49(18)	0.52^(1)^
Min-Max	11–152	27–182	
pNN50			
Median (IQR)	5.1(23)	10.7(18.9)	0.80^(1)^
Min-Max	0.8–68	0.3–62.9	
BMI z score			
Mean (SD)	2.5(0.3)	2.5(0.4)	0.97^(2)^
Min-Max	1.7–3.2	1.6–3.6	
LDL -cholesterol			
Median (IQR)	123(59.2)	98.8(50.3)	0.043*^(1)^
Min-Max	54.4–140	52–129.9	
Total Cholesterol			
Median (IQR)	180(27)	171(20)	0.098^(1)^
Min-Max	102–199	111–198	
TG			
Mean (SD)	102.5(40.5)	88.5(183)	0.009^*(2)^
Min-Max	65–129	62–139	
HDL			
Median (IQR)	41(11)	45(8)	0.01*^(1)^
Min-Max	28–50	27–56	
**Heart rate variability and lipid profile parameters at the end of the study (after supplementation of Omega-3)**			
RMSSD			
Mean (SD)	56.3(29)	45.6(29.8)	0.16^(2)^
Min-Max	10–140	10–140	
SDDN			
Mean (SD)	68(26.2)	59.6(29.5)	0.24^(2)^
Min-Max	29–143	27–156	
pNN50			
Mean (SD)	19.8(14.6)	15.6(14.8)	0.27^(2)^
Min-Max	1.1–59.1	0.9–60.1	
BMI z score			
Mean (SD)	2.5(0.33)	2.4(0.37)	0.46^(2)^
Min-Max	1.9–3.1	1.6–3.4	
LDL -cholesterol			
Mean (SD)	84.4(25)	77.7(23.6)	0.28^(2)^
Min-Max	29–143	52–125.5	
Total Cholesterol			
Mean (SD)	154.3(23.5)	152.9(18.9)	0.79^(2)^
Min-Max	98–179	110–179	
TG			
Mean (SD)	75.2(44)	74.8(29)	0.93^(2)^
Min-Max	42–110	49–130	
HDL			
Mean (SD)	43.9(61)	46.1(6.7)	0.18^(2)^
Min-Max	32–56	30–55	

*RMSSD* root mean square of successive differences, *SDDN* standard deviation of normal to normal R-R intervals, *pNN50* percentage of differences between adjacent normal heartbeats (NN intervals) that are greater than 50 ms, *LDL* low-density lipoprotein, *HDL* high-density lipoprotein, *TG* triglycerides, *IQR* interquartile range, *Min* minimum, *Max* Maximum, *SD* standard deviation, *BMI* body mass index.

*P value is considered significant if less than 0.05.

^(1)^Mann–Whitney test.

^(2)^Independent Samples *t* Test.

**Fig. 2:**
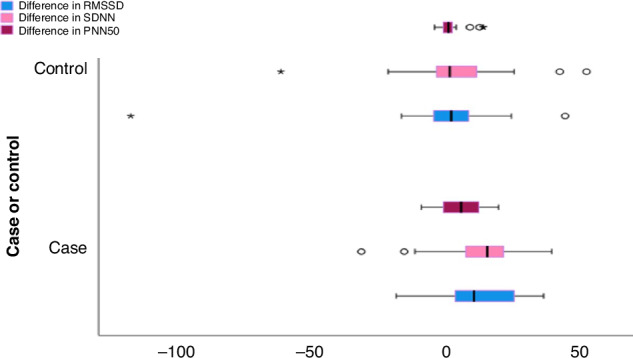
Cluster Box plot showing that the difference in the cases were more positive than in the control group.

The difference in heart rate variability parameters at the start of the study and after 3 months in each interventional (cases) and control group was calculated. This was done to study the effect of Omega-3 supplementation, and also to compare both groups simultaneously as shown in Table 3 and Figure 2. It seems that measures of heart rate variability increased significantly more in the interventional group than in the control group.

**Table 3:** Difference in measures of Heart rate variability: Post Omega -3 minus Pre Omega--3.

Measures of heart rate variability	Interventional (Cases)	Control	*P* value
Difference in RMSSD			0.017*^1^
Median (IQR)	10(23.7)	1.5(13)	
Min: Max	−19:36	−118:44	
Difference in SDNN			0.009^*1^
Median (IQR)	15 (14.25)	1 (15.7)	
Min-Max	−32:39	−62:52	
Difference in pNN50			0.043^*1^
Median (IQR)	5.2 (13.5)	0.5(3.4)	
Min-Max	−9.6:19.2	−4.7:13.6	

*IQR* interquartile range, *Min* minimum, *Max* maximum.

*P value is considered significant if less than 0.05.

^(1)^Mann–Whitney test; RMSSD: Root mean square of successive differences; SDDN: standard deviation of normal to normal R-R intervals; pNN50: percentage of differences between adjacent normal heartbeats (NN intervals) that are greater than 50 ms.

**Fig. 3:**
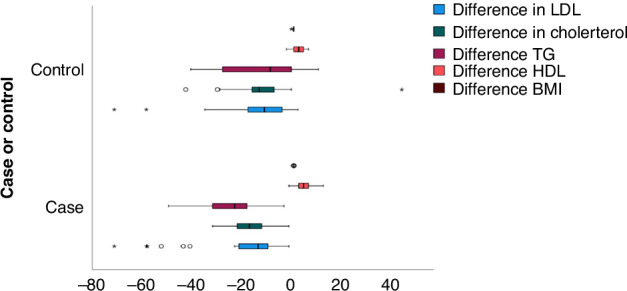
Cluster Box plot showing that the difference in the cases where more negative than in the control group as regards TG and more positive as regards HDL. The comparison of BMI may be very minimal to be seen.

Likewise, the difference in lipid profile parameters including BMI at the start of the study and after 3 months in each interventional (cases) and control group was calculated. This was done to study the effect of omega-3 supplements and compare both groups simultaneously as shown in Table 4 and Figure 3. Notably, triglyceride levels decreased significantly in the interventional group than in controls. On the other hand, HDL increased significantly in the interventional group more than in the control group.

**Table 4:** Difference in measures of lipid profile: Post Omega -3 minus Pre Omega—3.

Measures of lipid profiles	Interventional (Cases)	Control	*P* value
**The difference in LDL cholesterol**			0.151^1^
Median (IQR)	−14(12.3)	−11.9(16.6)	
Min: Max	−73:−2	−73:−1.7	
**The difference in total cholesterol**			0.102^1^
Median (IQR)	−18 (10.25)	−14.2 (9.5)	
Min-Max	−33:−2	−44:44	
**The difference in TG**			0.006^*1^
Median (IQR)	−24 (15)	−9.5(28.5)	
Min-Max	−51:−4	−42:0	
**The difference in HDL**			0.005^*2^
Mean (SD)	4(2.9)	1.9 (2.5)	
Min-Max	−2:12	−3:6	
**The difference in BMI Z score**			0.122^1^
Median (IQR)	0.0 (0.06)	−0.03 (0.13)	
Min-Max	−0.12:0.16	−0.0.92:0.18	

*LDL* low-density lipoprotein, *HDL* high-density lipoprotein, *TG* triglycerides, *IQR* interquartile range, *Min* minimum, *Max* maximum, *SD* standard deviation, *BMI* body mass index.

*P value is considered significant if less than 0.05.

^(1)^Mann–Whitney test.

^(2)^Independent Samples *t* Test.

**Fig. 4:**
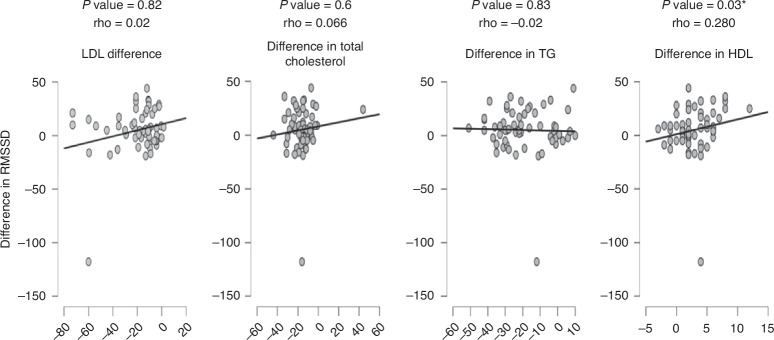
Correlations between RMSSD and laboratory lipid profiles. *P* value is considered significant if less than 0.05.

**Fig. 5:**
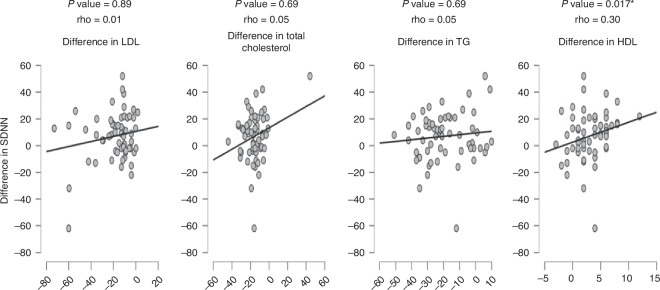
Correlations between SDNN and laboratory lipid profiles. *P* value is considered significant if less than 0.05.

**Fig. 6:**
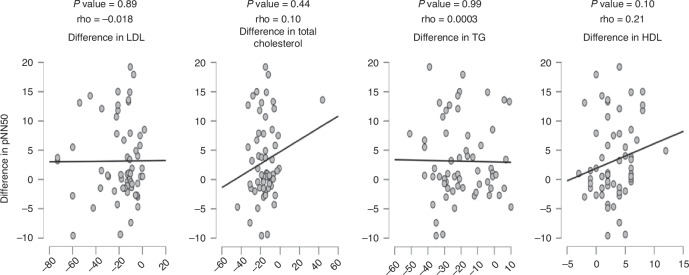
Correlations between pNN50 and laboratory lipid profiles. *P* value is considered significant if less than 0.05.

We were interested in disclosing if there are correlations between changes in measures of heart rate variability and laboratory lipid profiles. Spearman’s correlation was carried out that showed only significant positive, yet weak correlations between each of RMSSD, SDDN, and levels of HDL as shown in Figs. 4, 5, 6.

Regression model is done for each change in the lipid profile to control the basal lipid profiles as possible cofounders and it shows a significant decrease and a significant increase in differences in triglycerides and HDL respectively in the interventional group as referred and compared to the control group as shown in Table 5.

**Table 5:** Regression model to adjust variables that could affect changes in lipid profiles as the basal lipid profiles.

Studied variables	Univariable			Multivariable analysis	
	B	CI	*P* value	CI	*P* value
**Changes in LDL (post omega -pre omega)**					
Interventional versus control^a^	−5.15	−14.4: 4.16	0.25^b^	−9.40:6.99	0.770
Basal LDL	−0.33	−0.47: −0.19	<0.001	−0.47: −0.18	<0.001
**Changes in Total cholesterol (post omega -pre omega)**					
Interventional versus control^a^	−4.400	−10.38:1.58	0.146^b^	−8.20:2.21	0.254
Basal Total cholesterol	−0.246	−0.35: −0.14	<0.001	−0.34: −0.13	<0.001
**Changes in Triglycerides (post omega -pre omega)**					
Interventional versus control^a^	−12.31	−19.4: −5.16	0.001	−17.8: −2.8	**0.008**
Basal Triglycerides	−0.243	−0.43: −0.04	0.016	−0.34: 0.046	0.130
**Changes in HDL (post omega – pre omega)**					
Interventional versus control^a^	2.100	0.675:3.52	0.005	−0.25: −0.066	**0.001**
Basal HDL	−0.190	−0.28: −0.09	<0.001	0.035:2.78	0.045

*CI* confidence interval, *CI* Confidence interval.

^a^The control is the reference.

^b^In multivariable analysis, it is recommended to inflate the cutoff P value of univariable analysis to 0.2 or 0.25. It is usually not recommended to select the variables based on the cutoff of *P* < 0.05 because it may be possible that a variable individually insignificant but in a multivariable setup is significant or vice versa.^29^

**Fig. 7:**
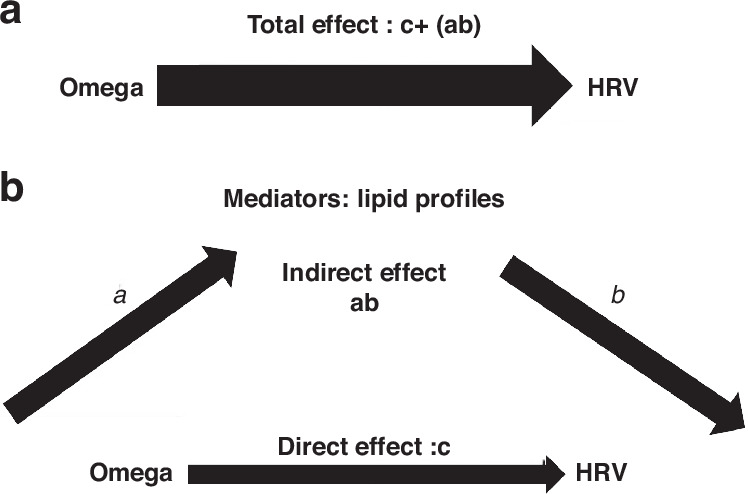
Path diagram for the mediation analysis. **a** The effect of omega on lipid profiles (path from omega to lipid profiles). **b** The effect of lipid profiles on HRV (the path from lipid profiles to HRV).

To prove the direct effect of omega on HRV, a mediation analysis was carried out to discover if the improvement of HRV is solely due to omega effect rather than improvement in lipid profiles as shown in Tables 6, 7, 8. The mediation analysis revealed that the direct effect of omega was predominated and changes in the lipid profiles exhibited negligible mediations on HRV. Figure 7 illustrates simply the path diagram of the mediation analysis.

**Table 6:** Mediation analysis for RMSSD

The outcome is improvement in RMSSD					
A.Direct effect of Omega supplementation					
Estimate	Std.Error	z-value	*P value	95% CI Lower level	95% CI Upper level
12.18	6.08	2	0.045	0.25	24.11
**B. Indirect effect: in the presence of mediators**					
**Mediator 1: Changes in LDL (post omega – pre omega)**					
−1.54	1.54	−0.97	0.33	−4.6	1.55
**Mediator 2: Changes in cholesterol (post omega – pre omega)**					
−1.04	1.24	−0.84	0.40	−3.49	1.39
**Mediator 3: Changes in triglycerides (post omega – pre omega)**					
0.37	2.46	0.15	0.87	−4.4	5.21
**Mediator 4: Changes in HDL (post omega – pre omega)**					
1.32	2.10	0.63	0.52	−2.79	5.44
**C.Total effect: Direct + Indirect**					
11.30	5.37	2.1	0.036	0.76	21.83

*CI* confidence interval.

*P value is considered significant if less than 0.05.

**Table 7:** Mediation analysis for SDNN.

The outcome is improvement in SDNN					
A.Direct effect of Omega supplementation					
Estimate	Std.Error	z-value	*P value	95% CI lower level	95% CI upper level
11.52	4.84	2.37	0.017	2.02	21.02
**B.Indirect effect: in the presence of mediators**					
**Mediator 1: Changes in LDL (post omega – pre omega)**					
−0.58	0.81	−0.71	0.47	−2.19	1.08
**Mediator 2: Changes in cholesterol (post omega – pre omega)**					
−2.05	1.59	−1.28	0.199	−5.19	1.07
**Mediator 3: Changes in triglycerides (post omega – pre omega)**					
−1.95	2.04	−0.95	0.33	−5.9	2.04
**Mediator 4: Changes in HDL (post omega – pre omega)**					
2.30	1.80	1.27	0.202	−1.23	5.85
**C.Total effect : Direct + Indirect**					
9.23	4.49	2.05	0.040	0.42	18.04

*CI* confidence interval.

*P value is considered significant if less than 0.05.

**Table 8:** Mediation analysis for pNN50.

The outcome is improvement in pNN50					
A.Direct effect of Omega supplementation					
Estimate	Std.Error	z-value	*P value	95% CI lower level	95% CI upper level
3.78	1.89	1.99	0.046	0.065	7.50
**B. Indirect effect: in the presence of mediators**					
**Mediator 1: Changes in LDL (post omega – pre omega)**					
0.07	0.25	0.27	0.78	−4.30	0.57
**Mediator 2: Changes in cholesterol (post omega – pre omega)**					
−6.36	0.53	−1.19	0.23	−1.67	0.40
**Mediator 3: Changes in triglycerides (post omega – pre omega)**					
−0.32	0.77	−0.41	0.67	−1.83	1.19
**Mediator 4: Changes in HDL (post omega – pre omega)**					
0.66	0.67	0.97	0.32	−0.66	1.99
**C. Total effect: Direct + Indirect**					
3.55	1.65	2.14	0.032	0.30	6.80

*CI* confidence interval.

*P value is considered significant if less than 0.05.

## Discussion

### Analysis of the finding

Omega supplementation showed a positive impact on the time domain heart rate variability as regards RMSSD, SDNN, and pNN50. In addition, triglycerides and HDL levels decrease and increase respectively and significantly in the interventional group receiving omega. Regarding the correlation between HRV and HDL, RMSSD and SDDN correlate positively with levels of HDL.

### Interpretation of findings

Omega and time domain HRV

Our study was carried out on overweight and obese children to outline the impact of omega-3 supplements on the variability of the heart rate. Heart rate variability is a normal phenomenon. The heart rate and its variations are the consequences of many impacts on the sinus node location.^21^ Omega-3 may have an obvious influence on voltage-gated ion channels of the sinus node region.^22^ Former research on the heart rate of recipients of cardiac transplants before and later to omega-3 treatment backs up this idea. It was observed that omega-3 supplementation lowers the heart rate in recipients of cardiac transplants who lack the vagal innervation for the cardiac muscle.^23^ These results propose that omega-3 can alter the conduction system’s electrophysiology of the heart. Omega-3 has the potential to lower the intrinsic heart rate of overweight and obese children via this route. Heart rate computed from cardiac beat-to-beat intervals (RR intervals) has a negative correlation with the heart rate variability due to the nonlinear connection between the two variables. As a result, a lower heart rate would be accompanied by increased heart rate variability.^24^ For all time domain metrics, higher values are likely to indicate a robust parasympathetic tone or low sympathetic activity. In the present report, we found that omega-3 fatty acid supplementation improved time domain metrics in overweight and obese children; the differences in all measures in heart rate variability in our study were increased positively more in the interventional than in controls, reflecting the cardioprotective effect of omega-3 supplementation.

#### Omega and lipid profiles

It is worth mentioning that the lipid profiles including triglycerides and HDL were more risky in cases than in the control group before omega-3 supplementation. However, omega-3 supplementation has reversed the situation. We can see that the differences in the levels of triglycerides and HDL between the end and the start of the study were decreased more and increased more respectively. These results may reflect the positive impact of omega-3 supplementation on the metabolic state in such susceptible overweight and obese children.

#### Correlations between time domain HRV and HDL

We observed a weak positive correlations between changes in RMSSD and SDDN, and changes in HDL levels. This observation agrees with a former research through which was concluded that patients with lower HDL levels may be prone to autonomic imbalances.^25^

### Strength of the study

This study may be the first study that investigated the impact of omega-3 as a cardioprotective represented in its effect on heart rate variability and the lipid profile simultaneously.

### Study limitations

The effect of omega-3 supplementation on BMI may not be clear due to the short time of this study. Future cohort research with a longer time may disclose such the proposed effect. However, BMI was maintained (and not increased) during the study period and this may be in accordance with the weight maintenance program (https://www.mayoclinic.org/diseases-conditions/childhood-obesity/diagnosis-treatment/drc-20354833) and this may be in accordance with the stepwise approach to the prevention and management of overweight and obesity in children.^26^ Therefore, omega -3 supplementation may have a balancing effect on BMI. Yet this suggestion should be further investigated.

Moreover, other measures of insulin sensitivity as fasting insulin and glucose weren’t available in the study setting. However, there are further measures for insulin sensitivity that we examined as lipid profiles and HRV measures.^27,28^

## Conclusion

Our data suggested that omega-3 supplementation may benefit cardiovascular health in overweight and obese children. The detected increases in the indices of the heart rate variability in our study may be beneficial because low heart rate variability indices and high heart rate have been linked to cardiovascular disease and a variety of comorbidities associated with obesity. In addition, our study may recommend screening children with lower HDL levels for autonomic imbalances represented in HRV.

## Supplementary information

Consort for the study

## Data Availability

The datasets generated during and/or analyzed during the current study are available from the corresponding author on reasonable request.
