# Supplementary material for: Omega and heart rate variability in overweight and obese schoolchildren
Source: Pediatr Res. 2025 Feb 19;98(3):957–65. doi: 10.1038/s41390-025-03913-5 (PMC12507677; doi:10.1038/s41390-025-03913-5)
Supplement: Supplementary file 1 — Consort for the study [file 41390_2025_3913_MOESM1_ESM.pdf]

CONSORT checklist of information to include when reporting a randomized trial*

Section/Topic	Item No	Checklist item	Reported on page No

Consolidated Standards of Reporting Trials

Title and abstract

	1a	Identification as a randomized trial in the title	1
	1b	Structured summary of trial design, methods, results, and conclusions (for specific guidance see CONSORT for abstracts)	1-3
**Introduction**			
Background and objectives	2a	Scientific background and explanation of the rationale	3-5
	2b	Specific objectives or hypotheses	1
**Methods**			
Trial design	3a	Description of trial design (such as parallel, factorial) including allocation ratio	5-8
	3b	Important changes to methods after trial commencement (such as eligibility criteria), with reasons	N/A
Participants			
	4a	Eligibility criteria for participants 5-6	
	4b	Settings and locations where the data were collected 5-8	
Interventions	5	The interventions for each group with sufficient details to allow replication, including how and when they were actually administered 5-8	

Outcomes	6a	Completely defined pre-specified primary and secondary outcome measures, including how and when they were assessed 6
8	6b	Any changes to trial outcomes after the trial commenced, with reasons : No changes
Sample size	7a	How sample size was determined 9
	7b	When applicable, explanation of any interim analyses and stopping guidelines: Not applicable
Randomization:		
Sequence generation	8a	The method used to generate the random allocation sequence: pages 5-8
	8b	Type of randomization; details of any restriction (such as blocking and block size) : Random allocation The
Allocation concealment mechanism	9	mechanism used to implement the random allocation sequence (such as sequentially numbered containers), describing any steps taken to conceal the sequence until interventions were assigned : software randomization
Implementation	10	Who generated the random allocation sequence : An independent person
		, who enrolled participants: The investigator
		and who assigned participants to interventions: An independent person
Blinding	11a	If done, who was blinded after assignment to interventions (for example, participants, care providers, those

		assessing outcomes) and how : those assessing outcomes
	11b	If relevant, description of the similarity of interventions: **not relevant**
Statistical Methods	12a	Statistical methods used to compare groups for primary and secondary outcomes : page 10
	12b	Methods for additional analyses, such as subgroup analyses and adjusted analyses : page10
**Results**		
Participant flow (a diagram is strongly	13a	For each group, the number of participants who were randomly assigned received the intended treatment and were analyzed for the primary outcome:**30 per group**

recommended) 13b For each group, losses and exclusions after randomization, together with reasons : No losses

Recruitment 14a Dates defining the periods of recruitment and follow-up : 3-4 and explained in the flow chart

14b Why the trial ended or was stopped : **Completion of the required sample size and required follow up**

Baseline data 15 A table showing baseline demographic and clinical characteristics for each group: **Table 1**

Numbers analyzed 16 For each group, number of participants (denominator) included in each analysis and whether the analysis was by original assigned groups : yes

Outcomes and estimation	17a	For each primary and secondary outcome, results for each group, and the estimated effect size and its precision (such as 95% confidence interval) : Page 7 and 8
	17b 18	For binary outcomes, the presentation of both absolute and relative effect sizes is recommended :**NA**
Ancillary analyses		Results of any other analyses performed, including subgroup analyses and adjusted analyses, distinguishing pre-specified from exploratory :**done**
Harms	19	All important harms or unintended effects in each group (for specific guidance see CONSORT for harms) :**Not applicable**

Discussion

Limitations	20	Trial limitations, addressing sources of potential bias, imprecision, and, if relevant, the multiplicity of analyses : **Not applicable**
Generalisability	21	Generalisability (external validity, applicability) of the trial findings : **A study is externally valid**
Interpretation	22	Interpretation consistent with results, balancing benefits and harms, and considering other relevant evidence **yes**

Other information

Registration	23	Registration number and name of trial registry : ***https://clinicaltrials.gov/study/NCT06555705: Effect of Omega 3 Supplementation on the Heart Rate Variability in Obese Children***
Protocol	24	Where the full trial protocol can be accessed, if available: **on ClinicalTrial.gov**
Funding	25	Sources of funding and other support (such as the supply of drugs), role of funders : **Self-Funded**
